# Fractionation and Characterization of Metallic Elements in Soils in Land Use Systems

**DOI:** 10.3390/toxics12020110

**Published:** 2024-01-28

**Authors:** Farid Ul Haq, Faridullah Faridullah, Muhammad Irshad, Aziz Ur Rahim Bacha, Farhan Hafeez, Zahid Ullah, Akhtar Iqbal, Awais Arifeen, Iqra Nabi, Abdulwahed Fahad Alrefaei, Mikhlid H. Almutairi

**Affiliations:** 1 Department of Environmental Sciences, COMSATS University Islamabad, Abbottabad Campus, Abbottabad 22060, Pakistanmirshad@cuiatd.edu.pk (M.I.);; 2 State Key Laboratory of Urban Water Resource and Environment, Shenzhen Key Laboratory of Organic Pollution Prevention and Control, School of Civil and Environmental Engineering, Harbin Institute of Technology Shenzhen, Shenzhen 518055, China; niqra18@fudan.edu.cn; 3 State Key Laboratory of Biogeology and Environmental Geology, School of Environmental Studies, China University of Geosciences, Wuhan 430074, China; 2201890048@cug.edu.cn; 4 Department of Zoology, College of Science, King Saud University, P.O. Box 2455, Riyadh 11451, Saudi Arabia; afrefaei@ksu.edu.sa (A.F.A.); malmutari@ksu.edu.sa (M.H.A.)

**Keywords:** metallic elements, land use systems, permafrost, pasture, forest, agricultural soils

## Abstract

Land use has a great impact on soil dynamics. The soils of various land use systems in Central Karakoram have been under immense pressure in the recent past due to certain anthropogenic activities such as land use practices and land use cover changes. These influences have an impact on the spatial distribution of metallic elements (MEs) in the soils of various land uses. Herein, we investigated the occurrence of the MEs, copper (Cu), zinc (Zn), and nickel (Ni), in soils of various land uses such as the permafrost, pasture, forest, and agricultural lands of the Central Karakorum region. The MEs were extracted in exchangeable, adsorbed, organically bound, carbonated, precipitated, and residual forms. The concentrations of MEs showed a significant dependence on the extraction method used, and the extraction trend followed the order of EDTA > HNO_3_ > KNO_3_ > NaOH > H_2_O. Zn showed the highest concentration compared to Ni and Cu in all extractions, whereas the land uses’ ME concentration followed the order of agricultural land > permafrost > forest > pasturelands. The highest values of total Zn, Ni, and Cu were 712 ± 01 mg/kg, 656 ± 02 mg/kg, and 163 ± 02 mg/kg, respectively, in agricultural soil. The ME concentration showed significant variations between different land uses, and the highest concentration was noted in agricultural soil. Zn was found to be a dominant ME compared to Ni and Cu. We believe this effort will provide opportunities for scholars to investigate MEs around the globe.

## 1. Introduction

Metallic elements (MEs) are influenced by edaphic processes as well as various anthropogenic activities [1]. Generally, environmental factors and catastrophic and natural events play an important role in land use and land cover changes (LULCCs), thus exposing less weathered elements to the topsoil. There is a significant effect of MEs on land use [2,3]; therefore, it is a globally adopted practice to document metallic elements’ basic data for their management [4,5,6,7]. Mountainous soils are fragile, and the main causes of spatial variability are various influencing environmental factors [8]. Parental substances are the primary source of MEs in soils and have a long-lasting impact on soil properties [9]. The altitudinal variations in mountain ranges like Central Karakorum also play a key role in the transportation of MEs in land use systems [10,11,12]. In such regions, the soils at high altitudes remain frozen. Permafrost is frozen ground remaining at or below 0 °C for at least two consecutive years [13].

Permafrost acts as sink for atmospheric carbon that may become an important source of greenhouse gases as a result of global warming [14]. At high altitudes, thawing of the permafrost results in the transfer of MEs to land uses like pastures, forests, and agricultural systems through precipitation and other environmental agents. Globally, pastures occupy 3.2 to 4.9 billion hectares of land [15]. Inhabitants of the study area are dependent on pastures, and they migrate uphill through pasture slopes with livestock in summer, which is the major source of income [16]. However, MEs in pastures are greatly influenced by human activities such as road infrastructure, accessibility, communication, and development [17]. There is also forest cover in Central Karakorum region with a diversity of plant species. According to the Food and Agricultural Organization (FAO) definition, forests are areas of land larger than 0.5 hectares that include trees that are at least 5 m tall and have a canopy covering more than 10% of the land or trees that can naturally attain these thresholds. Land that is primarily used for urban or agricultural purposes is not included. The higher dependence of humans on forests has adversely affected forest land use systems. The topsoil is generally more affected due to its vulnerability to anthropogenic inputs [18,19]. Forests attract tourists and local inhabitants. Tourist activities lead to contamination, which increases the MEs in soils of various land uses [20].

The application of fertilizers and urbanization both impact the ME and nutrient status of soils, especially in agricultural land use systems. Such accessible land use systems are more affected by human development. The land uses near roads are more prone to higher concentration of MEs [21,22,23,24], and increased traffic flow further enhances the concentrations in the vicinity [25,26]. Regardless of land use, the physicochemical properties of the soil influence the fertility and productivity of the soil. Soil physical properties affect the root penetration, water movement, availability, and retention of plant nutrients [27,28]. Soil chemical properties also influence the productivity of land because they influence the solubility and bioavailability of elements [29].

LULCCs have a long-lasting impact on the inhabitants and the environment as a whole [30]. Numerous studies have focused on ME dynamics in various types of land uses across the globe [31]. However, researchers have put less emphasis on economically marginal and ecologically vulnerable land uses like those in the Central Karakoram region. Limited studies have been conducted in mountainous areas, with a wide range of results [32]. In this study, we investigated the concentration of MEs, i.e., Cu, Zn, and Ni, in selected land uses in the Central Karakorum region. There is a need to explore the MEs dynamics in order to have an in-depth record of their spatial distribution. This study will provide researchers with basic knowledge of MEs in the Central Karakoram region and will also serve as a reference for researchers to understand ME mobility and dynamics under LULCCs.

## 2. Experimental Section

### 2.1. Area Description

The selected study area is located in the northern area of Pakistan in Gilgit Baltistan province, in the Central Karakoram region. The sites are along the China–Pakistan Economic Corridor (CPEC) that connects Pakistan with China through the Khunjarab Pass. Administratively, this area has three districts: Hunza, Nagar, and Gilgit. The selected research sites are the Gojal and Karimabad valleys in Hunza District, the Rakaposhi area in Nagar District, and the Jutial area, which is a town in Gilgit District. The Karakoram region stretches from the northwest and extends east toward China and India. The area has glaciers and snow that melt in the summers and feed various areas before reaching the Indus River.

**Figure 1:**
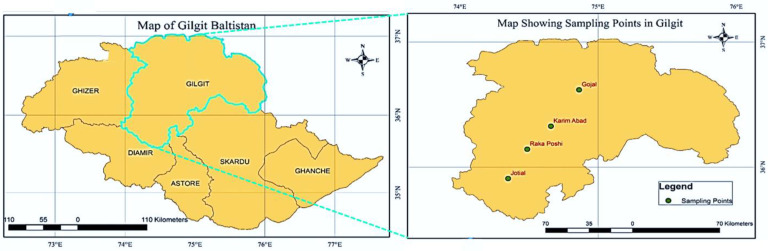
Study area map of Gilgit division and sampling points (Gojal, Karimabad, Rakaposhi, and Jotial).

There are extreme climatic conditions in the Gojal, Hunza, and Nagar districts in winter, while the climate in Gilgit is comparatively moderate. The maximum annual precipitation in the Hunza and Nagar valleys is 136.2 mm [33]. Hunza and Nagar are scarcely populated compared to Gilgit, which is densely populated and serves as the economic and administrative hub of the province. A significant part of this population is associated with agriculture and livestock, which is dependent upon the agricultural, forest, and pasture lands. The geographical location of the study area extends from 74°19′45″ E to 74°46′54″ E and 35°49′33″ N to 36°38′47″ N, with the lowest altitude of 1490 m up to approximately 4200 m above sea level (Figure 1).

### 2.2. Soil Sampling

A total of 64 soil samples were collected from topsoils (0–30 cm) randomly from Gojal Hunza, Karimabad Hunza, Rakaposhi Nagar, and Jutial Gilgit in four replications from four land uses i.e., permafrost, pasture, forest, and agricultural land use. The sites were selected in such a way that all the land uses fell along the same gradient in each area. The samples were taken to COMSATS University Islamabad (CUI) soil science laboratory for further analysis. The samples were ground into powder form and screened through a 2 mm dimensional sieve tube.

### 2.3. Physicochemical Properties Analysis

The moisture content was gravimetric. Using the dry combustion method, 20 g of soil was heated to 550 °C for 24 h. To determine the soil organic matter, the percentage difference between the original and end soil weight was calculated. The pH and EC were measured using the weight-to-volume ratio (*w*/*v*) of water and soil, which was 1:5 [34]. The pH and EC were measured by mixing 4 g of soil with 20 mL of deionized (DI) water, letting the mixture settle for 20 min, and then dipping the electrode of the pH/EC meter into the soil solution for 30 s. The soil texture was determined by the hydrometer method, and the soil textural class was investigated using the International Soil Science Society Classification System (ISSS) [35].

### 2.4. Metallic Elements (MEs) Fractionation

An adjusted variant of a successive extraction method [36] was used to fractionate the MEs. Successive extractions were conducted using 3 g of a sample in a 50 mL centrifuge tube. The fractionations of the selected MEs are replaceable, and adsorbed which can be naturally bound to carbonated precipitate while the obtained structure can play a role in successive extractions. First, 25 mL of 0.5 M potassium nitrate (KNO_3_) was added to the sample and shaken for 16 h. Then, 25 mL of DI water was added to the residue and shaken for 2 h; next, 25 mL of 0.5 M solution of sodium hydroxide (NaOH) was added to the residue and shaken for 16 h; then, 0.05 M of ethylenediaminetetraacetic acid disodium salt dihydrate (Na_2_EDTA) was added to the residual sample and shaken for 6 h. Finally, 4 M nitric acid (HNO_3_) was added to the residue and shaken for 16 h. The sample tube was spun for 10 min at a speed of 2500 rpm during each fraction. Subsequently, the supernatant was collected and separated by a 0.22 μm channel. The different forms were extracted as follows.

#### 2.4.1. Exchangeable Form

The MEs were easily swapped out for other ions in the soil solution because they have a weak binding to the soil particles, and they were identified by taking 0.5 M of a KNO_3_ solution, shaking and centrifuging it for 16 h, and then filtered.

#### 2.4.2. Adsorbed Fraction

MEs adsorbed on the soil surface or sediment particles are not firmly tied and are free under specific circumstances. MEs were determined by taking 25 mL of deionized water following the shaking and centrifugation for 2 h. The obtained mixture was filtered.

#### 2.4.3. Organic Bounded Fraction

MEs bonded to the sample’s organic substance make up this proportion. These fractions were determined by collecting the residues of the adsorbed fraction in 0.5 M NaOH, centrifuging and shaking them for 16 h, and then filtering them.

#### 2.4.4. Carbonate Precipitated Fraction

The carbonated precipitated form was investigated by taking residues of the organic bounded metals in 0.05 M Na_2_EDTA, shaking and centrifuging them for 6 h, and filtering them.

#### 2.4.5. Residual Form

MEs that are firmly bonded to the sample’s mineral matrix are relatively immobile and are difficult to release. Residual MEs were determined by collecting the residues of the carbonated precipitated metal in 4 M HNO_3_ following the shaking and centrifugation for 16 h at 80 °C, and then filtered.

### 2.5. Quality Control (QC)/Quality Assurance (QA)

Quality control (QC) measures were taken into consideration by using blanks, triplicate samples, and a standard reference material (SRM-2711). The ME, i.e., copper (Cu), zinc (Zn), and nickel (Ni), concentration was determined using an atomic absorption spectrometer (AAS Perkin Elmer A Analyst 700 made in the USA). The data were obtained with a standard nebulizer and flow spoiler. The standard calibration for Cu, Ni, and Zn was performed using an AAS with the wavelengths 324.8 nm, 232.0 nm, and 231.9 nm, respectively. The lower limits of detection for Cu, Ni, and Zn were 0.25, 0.30, and 0.15 mg/Kg, respectively. For the stock standard solution of Cu and Ni, 1 g of both (Cu and Ni metals) was dissolved in a minimum volume of (1 + 1) HNO_3_ diluted to 1 L with 1% (*v*/*v*) HNO_3_. For the stock standard solution of Zn, 5 g of Zn metal was dissolved in a minimum volume of (1 + 1) HCl diluted to 1 L with 1% (*v*/*v*) HCl. Three measurement readings on the AAS were used to obtain the mean value for each sample, and the standard deviation of the data was used to determine the error estimate [37]. The statistical analyses were performed by using the Analysis of Variance (ANOVA) and Pearson Correlation.

## 3. Results

Table 1 indicates the physio-chemical properties of the soil samples collected from various land uses. The pH values of the soil samples collected from Gojal Hunza were between 6.85 and 7.75. The pH values in Gojal Hunza varied as agricultural land > pastureland > permafrost > forest. The pH values of agricultural, pasture, permafrost, and forest soils in Gojal Hunza were observed to be 7.75, 7.55, 7.25, and 6.85, respectively. In Jutial Gilgit, the trend differed with the pH values in the following order: agricultural land > pastureland > permafrost > forest land. In Jutial Gilgit, the pH values for agricultural, pasture, forest, and permafrost soils were observed to be 7.45, 7.40, 6.75, and 6.20, respectively.

In Karimabad Hunza, the pH values followed the order: agricultural land > pastureland > permafrost > forest land. For Rakaposhi Nagar, the pH of the agricultural soil was 7.35, pastureland 7.55, forest soil 6.75, and permafrost soil 6.95. The highest EC was noted in the permafrost soil (538 µS/cm), while the lowest value was noticed in the pasture soils (384 µS/cm) of Gojal Hunza. The EC of agricultural land was 506 µS/cm followed by forest land with an EC of 444 µS/cm. In Jutial Gilgit, the highest EC was noticed in pastureland (489 µS/cm), while the lowest value was found in forest land (385 µS/cm). In Karimabad Hunza, the EC varied between 375 and 505 µS/cm for various land uses. The maximum moisture content (31.5%) was noted in the permafrost soil of Karimabad Hunza, while the minimum was noticed in the agricultural soil of Gojal Hunza. The soil moisture content varied in Gojal Hunza and Rakaposhi Nagar in the following order: permafrost soil > pasture soil > forest soil > agricultural land soil. However, the soil moisture content in Karimabad Hunza and Jutial Gilgit followed a different trend: permafrost soil > forest soil > pasture soil > agricultural soil.

Forest soil showed a maximum organic matter (OM) ranging from 2.45 to 2.85%. The OM trend in land uses of all regions varied as forest > agriculture > pasture > permafrost, except for Rakaposhi Nagar in which the OM in the pasture soil was slightly more than in the agricultural soils. The lowest amount of organic matter was observed in the permafrost soils ranging from 0.70% to 1.75%. The amount of organic matter in the pasture and forest soils was 1.65–2.55%, and 2.45–2.85%, respectively. The OM content in various areas was in the following order: Gojal Hunza > Jutial Gilgit > Rakaposhi Nagar > Karimabad Hunza. In terms of forest land use, the maximum OM was noted in Rakaposhi Nagar followed by Gojal Hunza and Jutial Gilgit, respectively, whereas Karimabad Hunza had the lowest OM. In pastureland, the OM followed the pattern: Rakaposhi Nagar > Karimabad Hunza > Gojal Hunza > Jutial Gilgit.

The concentration of MEs was highly dependent on the extraction method. The maximum concentration of MEs was noticed with Na_2_EDTA followed by HNO_3,_ KNO_3,_ and NaOH, and the lowest concentration of MEs was observed in adsorbed form (Table 2, Table 3 and Table 4). Two-way Analysis of Variance (ANOVA for statistical analysis), as represented by lowercase letters in superscript, indicates statistically significant differences among the various land uses in the same regions. Agricultural lands have comparatively more accumulation of MEs due to the frequent use of agrochemicals [38,39]. In this agricultural land, the application of fertilizers is a common practice for higher crop yield. The high Ni concentration in agricultural land uses may be due to the frequent use of fertilizers. Among all land uses, the maximum concentration of exchangeable Ni was noted in the agricultural soils of Karimabad Hunza (6.70 mg/kg), while the pasture soil of Gojal Hunza (1.25 mg/kg) showed the lowest concentration. MEs are present in relatively high concentrations in urban and agricultural soils as compared to other land uses, as agricultural land use is easily accessible to humans [40].

Table 2 indicates that the highest water-soluble Ni was observed in the agricultural soils of Rakaposhi Nagar (3.50 mg/kg), whereas the permafrost soils of Karimabad Hunza had the lowest value (0.30 mg/kg). Among all land use systems, the highest organically bound Ni was observed in the agricultural soils of Karimabad Hunza (8.95 mg/kg), while the lowest (3.35 mg/kg) concentration was in its pasture soils. Human-induced influences may increase the Ni in land uses that are easily available. The agricultural soil of Rakaposhi showed the highest value of Ni (373 mg/kg) in carbonated precipitated form, whereas the pasture soil of Karimabad Hunza showed the lowest value (207 mg/kg). Residual fractions of MEs are not readily available under normal conditions. However, the maximum residual Ni was found in the agricultural soil of Gojal Hunza (310 mg/kg), while the pasture of Karimabad had the lowest value (115 mg/kg) after extraction.

Table 3 indicates that for various extraction methods, the concentration of Cu in the soils of various land-use systems were in the following order: EDTA > HNO_3_ > KNO_3_ > NaOH > H_2_O. Generally, Cu is extensively used in electrical cables, various alloys, cooking utensils, chemical factories, fertilizers, and pesticides. The maximum plant-available Cu was observed in the agricultural soil of Karimabad Hunza (21.1 mg/kg), while the pasture soil of Gojal Hunza had the lowest amount of Cu (11.1 mg/kg). In water-soluble form, the highest Cu adsorption was found in the Gojal Hunza agricultural soils (1.70 mg/kg), while the lowest was observed in the Jutial Gilgit pasture soil (0.25 mg/kg). The highest value of organically bound Cu was noticed in Karimabad Hunza’s agricultural soils (16.2 mg/kg), and the lowest concentration was observed in the pastures of Gojal Hunza and Jutial Gilgit (11.3 mg/kg). The maximum amount of Cu was in carbonated precipitated form in the agricultural soils of Rakaposhi Nagar with the highest value of 97.7 mg/kg, whereas the lowest value was observed in Jutial Gilgit’s forest soil at 31.40 mg/kg. In residual form, Cu showed the highest concentration in the permafrost soil of Karimabad Hunza (30.8 mg/kg), and the lowest concentration was found in the forests of Jutial Gilgit (12.90 mg/kg).

Table 4 indicates the amount of Zn extracted from various land uses using different extraction methods. The concentration of Zn followed the order: EDTA > HNO_3_ > KNO_3_ > NaOH > H_2_O. The highest concentration of water-soluble Zn was observed in the agricultural soils of Rakaposhi Nagar (1.65 mg/kg), while Jutial Gilgit’s pasture soil had the lowest concentration (0.15 mg/kg). The highest value of organically bound Zn was found in the agricultural soil of Karimabad Hunza (24 mg/kg), and the lowest was found in the forests of Gojal Hunza (7.85 mg/kg). The agricultural soil of Gojal Hunza showed the highest concentration (336 mg/kg) in carbonated precipitated form, while the forest soil of Rakaposhi Nagar displayed the lowest concentration (102 mg/kg). The Ni extracted in residual form was highest in the agricultural soil of Gojal Hunza (298 mg/kg), while Jutial Gilgit’s pasture soil exhibited the lowest concentration (92.6 mg/kg), as demonstrated in Table 4.

**Figure 2:**
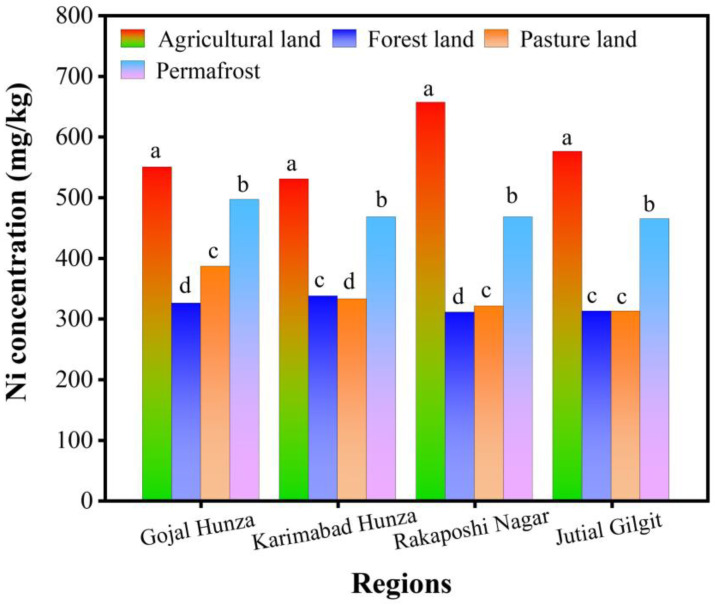
Total Ni concentration (mg/kg) in various land uses in Central Karakoram (the average data are presented here, while the letter represents the statistically significant Ni concentration among different regions).

**Figure 3:**
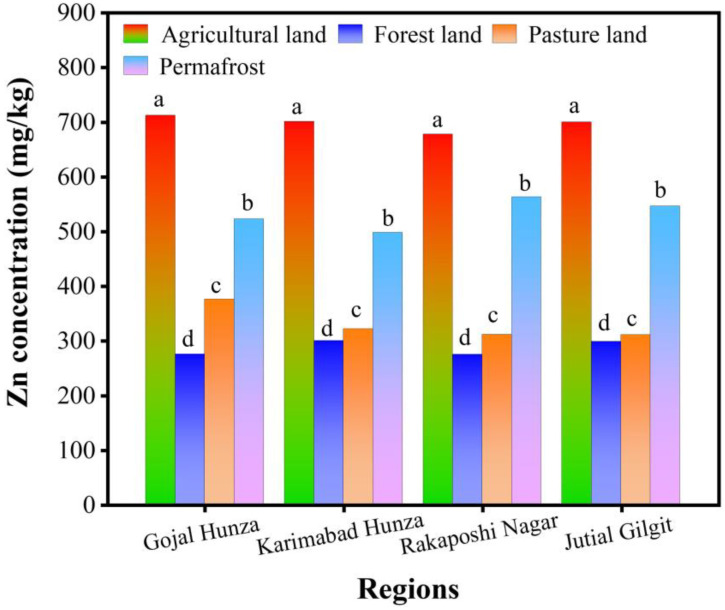
Total Zn concentration (mg/kg) in various land uses in Central Karakoram (the average data are presented here, while the letter represents the statistically significant Zn concentration among different regions).

**Figure 4:**
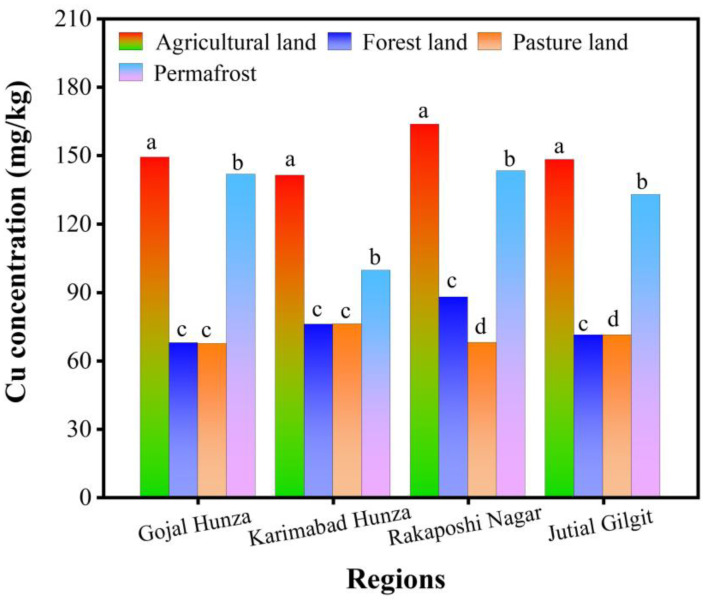
Total Cu concentration (mg/kg) in various land uses in Central Karakoram (the average data are presented here, while the letter represents the statistically significant Cu concentration among different regions).

Figure 2, Figure 3 and Figure 4 indicate the maximum concentration of MEs in total form in agricultural land use. Zn had the highest concentration in the soils, followed by Ni, whereas Cu was observed to have the lowest content in soils of all land use systems in total form. Figure 2 shows the highest concentration of total Ni in agricultural soils and the lowest in the pasture and forest soils of various land uses across all four regions.

Figure 4 shows that the highest concentration of Cu was detected in permafrost and agricultural soils, except in Karimabad Hunza, where the amount of Cu was comparatively lower. Figure 4 indicates the total Cu in all four land uses was in the following order: agricultural soil > permafrost soil > forest soil > pasture soil.

Table 5 displays the Pearson correlation coefficient among selected physicochemical properties and the total Ni, Zn, and Cu, as shown in Figure 2, Figure 3 and Figure 4. Generally, a non-significant correlation was noted among total MEs and physicochemical properties except in the Gojal Hunza and Rakaposhi Nagar regions. The pH presented a non-significant positive correlation with MEs in Gojal Hunza, Karimabad Hunza, and Jutial Gilgit. In the Rakaposhi Nagar region, Ni (r^2^ = −0.24, *p* = 0.76) and Zn (r^2^ = −0.175, *p* = 0.825) displayed a negative nonsignificant correlation with the pH. A significant positive correlation of EC was found between Ni (r^2^ = 0.99, *p* = 0.008) and Zn (r^2^ = 0.97, *p* = 0.034) in the Gojal Hunza region. EC showed a negative non-significant correlation with the MEs Ni (r^2^ = −0.16, *p* = 0.83) and Zn (r^2^ = −0.30, *p* = 0.698) in the Karimabad Hunza region. The soil MC displayed a positive non-significant correlation with all MEs across all regions, except for Cu in Karimabad Hunza and Jutial Gilgit, which was observed to have a negative non-significant correlation (r^2^ = −0.06, *p* = 0.945), (r^2^ = −0.91, *p* = 0.086). Cu, Ni, and Zn were observed to be negatively correlated with OM in all regions. Organic matter showed a significant negative correlation with Cu (r^2^ = −0.96, *p* = 0.039) and Ni (r^2^ = −0.96, *p* = 0.042) in the Rakaposhi Nagar region.

## 4. Discussion

Soil physio-chemical properties in land use systems form a strong relationship with the productivity and fertility of the soil by affecting the retention and infiltration of water that determines the bioavailability of the nutrients [41]. The pH values in Gojal Hunza were found to be higher in agricultural land. The high values of pH in agricultural land use are in agreement with the observations recorded in the Central Karakorum region [42]. However, these results also contradict the findings in land uses in Naltar Valley, where potato fields were acidic, as compared with forest and pasture land use [43]. Generally, the higher pH of soils is referred to as richer organic matter [44,45]. In Karimabad Hunza, the pH values in agricultural land were observed to be higher. The basic trend of pH in agricultural soil is in agreement with the observations of the agricultural land use system in Bagrote Valley, near the junction of the Himalayan and Karakorum ranges [46,47,48,49]. The EC values were found to be higher in agricultural land uses across all the regions in Central Karakorum. A higher value of EC in arable soil could be due to salts in the soil or the use of chemical fertilizers [50]. The highest moisture content was noted in the permafrost soil across the selected regions of Central Karakorum, and the lowest was recorded in agricultural soils, which is in agreement with the findings of many researchers [51]. Soil moisture content is one of the most important parameters for vegetation, and organic carbon is a major input for soil [41]; however, it may evaporate readily. Generally, a higher OM was observed in forest land use. The results agree with the results observed in mid-hill Nepal, where soil organic matter was found to be higher in the forests than in agricultural land [41]. Due to the thawing in summer, the organic matter may either leach down or may be transported from the higher altitudes to lower altitudes along with the topographical gradient. The higher organic matter content in forests is due to the plants, leaves, and branches; nevertheless, the low organic matter in pastures is due to the overgrazing of livestock and wildlife, which is in agreement with previous studies [52]. In general, the physiochemical properties have a strong influence on various land use systems [46,47,48,49].

The main purpose of the fractionation was to study the concentration and dynamics of metallic elements (MEs) in four land use systems. The land use effect on the ME dynamics was prominently visible, as the agricultural land use showed a higher concentration of MEs, suggesting anthropogenic inputs. As mentioned in Table 2, Table 3 and Table 4, the sequential fractionation also revealed the elevated concentration of MEs in some permafrost soils (possibly suggesting the presence of MEs in the parent materials), which may be investigated as another aspect of the study. The pastures and forests showed comparatively lower amounts of MEs, which may be due to the leaching and drainage of MEs from these land uses with precipitation and other environmental factors, as pastures and forests are at higher altitudes than agricultural land use systems.

Generally, the selected metallic elements were found to be higher in agricultural land use than in other land use systems. One of the reasons for the elevated amount of metallic elements may be the frequent use of agrochemicals [38,39]. In Central Karakoram, there are small leveled agricultural land patches commonly irrigated by glacial water and water channels for crop production. In these agricultural land uses, there is a tendency toward the use of chemical and organic fertilizers for increased crop yields. HMs are present in relatively high concentrations in urban and agricultural soils as compared to other land uses like forests, as agricultural land use is easily accessible to humans [40].

Cu is extensively used in electrical cables, various alloys, cooking utensils, chemical factories, fertilizers, and pesticides. The highest content of Cu was observed in the agricultural soil of Karimabad Hunza. The elevated amount of Cu in agricultural land use supports various findings of researchers, as fertilizers for agriculture practice increase the concentration of Cu in soil [53]. The elevated concentration of Cu in agricultural soil is a sign of the long-term intensive use of fertilizers and pesticides [54]. One of the drawbacks of elevated values of the MEs is the fact that increased concentrations of MEs like Cu and Zn can affect the uptake of other essential nutrients by plants [55]. Some soils have more affinity to adsorb elements like Cu and Zn, which are used in the form of fertilizers due to the functional groups of organic compounds [56].

## 5. Conclusions and Recommendations

There are many dominating factors for change in the status of MEs in soils of various land uses in mountainous areas like Central Karakoram. Among the human-induced impacts on land use systems are commercial and economic activities due to rapid urbanization and the boom in the tourism and hospitality industry. However, the parent material also has an influence on the spatial distribution of MEs in the soils. It was observed that the concentrations of MEs in land use systems such as permafrost, forest, pasture, and agricultural lands varied significantly. Generally, the trend in terms of the amount of MEs under various land uses was EDTA > HNO_3_ > KNO_3_ > NaOH > H_2_O irrespective of the land uses. Agriculture soils were observed to have higher ME concentrations in all the selected areas. In a comparison of MEs, Zn was observed to have the highest concentration in the soils followed by Ni, whereas Cu had the lowest content. This research indicates that the concentration of MEs was significantly high in agricultural soils as compared to other land use types. Some recommendations are proposed, keeping in view the future prospects of this work.

- The qualitative productivity of soils should be protected so that the ecological functioning is not affected.
- The use of land may carefully be rationalized according to its capacity by devising land use management practices for its sustainability.
- The conservation and enhancement of soil fertility may be achieved through long-term management policies under government patronage.
- Future industrialization and urbanization may carefully be planned in the area keeping under consideration the ecological risk associated with MEs.
- The Central Karakorum region is highly prone to climate change; therefore, initiatives for combating climate change should be undertaken both nationally and internationally, as it directly affects the land use system.

## Figures and Tables

**Table 1:** Physio-chemical properties in various land uses in Central Karakoram.

Land Uses	Regions															
	Gojal Hunza				Jutial Gilgit				Karimabad Hunza				Rakaposhi Nagar			
	pH	EC	Moisture Content (%)	Organic Matter (%)	Ph	EC	Moisture Content (%)	Organic Matter (%)	pH	EC	Moisture Content (%)	Organic Matter (%)	pH	EC	Moisture Content (%)	Organic Matter %
Agricultural land	7.75± 0.06	506± 4	18.5± 0.70	2.35± 0.04	7.45± 0.06	470± 2	20.5± 0.70	2.35± 0.04	7.25± 0.08	505± 3	22.5± 1.0	2.15± 0.04	7.35± 0.03	525± 4	22.5± 0.85	2.25± 0.06
Forest land	6.85± 0.07	444± 4	23± 1.41	2.65± 0.05	6.75± 0.07	385± 4	26.5± 0.85	2.55± 0.03	6.35± 0.04	461± 4	28.5± 0.82	2.45± 0.08	6.75± 0.02	443± 7	27.5± 0.90	2.85± 0.04
Pastureland	7.55± 0.05	384± 7	23.5± 0.71	1.69± 0.03	7.4± 0.00	489± 4	25.5± 0.71	1.65± 0.07	6.95± 0.06	400± 4	27.5± 0.45	1.85± 0.05	7.55± 0.04	469± 4	28.5± 0.65	2.55± 0.03
Permafrost	7.25± 0.03	538± 1	31± 1.41	0.85± 0.07	7.2± 0.02	435± 3	29.00 ±0.00	0.7±. 02	6.65± 0.07	375± 4	31.5± 0.70	1.25± 0.07	6.95± 0.06	484± 3	32.5± 0.70	1.75± 0.05

**Table 2:** Ni concentration in various land uses in Central Karakoram using different extraction methods.

Regions	Land Uses	Ni Concentration Using Different Extraction Methods				
		KNO_3_ (mg/kg)	H_2_O (mg/kg)	NaOH (mg/kg)	Na_2_EDTA (mg/kg)	HNO_3_ (mg/kg)
Gojal Hunza	Agricultural land	5.5 ± 0.85 ^a^	1.2 ± 0.42 ^b^	8.75 ± 0.49 ^a^	313 ± 0 ^b^	310 ± 1 ^a^
	Forest land	2.85 ± 0.07 ^b^	1.4 ± 0.28 ^a^	3.95 ± 0.78 ^b^	216 ± 1 ^c^	130 ± 1 ^c^
	Pastureland	1.25 ± 0.07 ^c^	0.45 ± 0.49 ^c^	3.95 ± 0.21 ^b^	206 ± 2 ^d^	122 ± 1 ^c^
	Permafrost	3.3 ± 0.14 ^b^	1.35 ± 0.07 ^a^	8.5 ± 0.85 ^a^	336 ± 0 ^a^	144 ± 0 ^b^
Karimabad Hunza	Agricultural land	6.7 ± 0.28 ^a^	3.1 ± 0.42 ^a^	8.95 ± 0.78 ^a^	316 ± 0 ^b^	285 ± 1 ^a^
	Forest land	7 ± 0.28 ^a^	1.4 ± 0.28 ^b^	5.25 ± 0.92 ^b^	216 ± 1 ^c^	129 ± 1 ^c^
	Pastureland	3.05 ± 0.64 ^b^	2.8 ± 0.42 ^a^	3.35 ± 0.64 ^c^	207 ± 0 ^d^	115 ± 2 ^d^
	Permafrost	2.75 ± 0.92 ^b^	0.3 ± 0.14 ^c^	7.5 ± 0.99 ^a^	355 ± 0 ^a^	156 ± 0 ^b^
Rakaposhi Nagar	Agricultural land	5.1 ± 0.28 ^a^	3.5 ± 0.99 ^a^	8.5 ± 0.57 ^a^	373 ± 1 ^a^	260 ± 0 ^a^
	Forest land	4.35 ± 0.35 ^b^	1.05 ± 0.21 ^c^	4.75 ± 1.20 ^b^	233 ± 0 ^c^	119 ± 0 ^c^
	Pastureland	3.0 ± 0.14 ^c^	3.45 ± 0.35 ^a^	6.55 ± 0.92 ^a^	218 ± 1 ^d^	116 ± 1 ^d^
	Permafrost	3.8 ± 0.42 ^c^	2.45 ± 0.49 ^b^	7.45 ± 0.92 ^a^	343 ± 0 ^b^	132 ± 1 ^b^
Jutial Gilgit	Agricultural land	5.65 ± 0.64 ^a^	3.15 ± 0.49 ^a^	8.5 ± 0.85 ^a^	361 ± 1 ^a^	281 ± 1 ^a^
	Forest land	3.35 ± 0.64 ^c^	1.35 ± 0.35 ^b^	4.2 ± 0.57 ^b^	231.8 ± 1 ^c^	121 ± 0 ^c^
	Pastureland	3.15 ± 0.35 ^c^	3.8 ± 0.42 ^a^	5.55 ± 0.49 ^b^	220.05 ± 1 ^d^	119 ± 0 ^c^
	Permafrost	3.85 ± 0.07 ^b^	2.6 ± 0.28 ^a^	7.5 ± 0.42 ^a^	342.3 ± 1 ^b^	138 ± 1 ^b^

Significant differences in various land uses are shown by letters in superscript.

**Table 3:** Cu concentration in various land uses of Central Karakoram using different extraction methods.

Regions	Land Uses	Cu Concentration Using Different Extraction Methods				
		KNO_3_ (mg/kg)	H_2_O (mg/kg)	NaOH (mg/kg)	Na_2_EDTA (mg/kg)	HNO_3_ (mg/kg)
Gojal Hunza	Agricultural land	19.4 ± 0.2 ^a^	1.7 ± 0.14 ^a^	13.8 ± 0.1 ^a^	90.0 ± 0.7 ^a^	23.15 ± 0.3 ^a^
	Forest land	11.6 ± 0.1 ^c^	0.5 ± 0.14 ^b^	12.3 ± 0.1 ^b^	32.5 ± 1.5 ^d^	13.7 ± 0.2 ^b^
	Pastureland	11.1 ± 0.2 ^d^	0.45 ± 0.07 ^c^	11.3 ± 0.1 ^c^	35.2 ± 0.6 ^c^	20.7 ± 0.4 ^a^
	Permafrost	16.5 ± 0.2 ^b^	0.7 ± 0.14 ^b^	14.0 ± 0.6 ^a^	85.4 ± 0.1 ^b^	22.9 ± 0.2 ^a^
Karimabad Hunza	Agricultural land	21.1 ± 0.4 ^a^	1.25 ± 0.21 ^a^	16.2 ± 0.4 ^a^	94.4 ± 0.4 ^a^	32.5 ± 2.8 ^a^
	Forest land	12.6 ± 0.2 ^c^	0.4 ± 0.14 ^c^	11.9 ± 0.3 ^c^	31.2 ± 0.5 ^d^	14.2 ± 0.9 ^b^
	Pastureland	12.4 ± 0.3 ^d^	0.25 ± 0.07 ^d^	12.4 ± 0.1 ^c^	33.5 ± 0.1 ^c^	14.6 ± 0.4 ^b^
	Permafrost	17.9 ± 0.3 ^b^	0.65 ± 0.07 ^b^	15.1 ± 0.2 ^b^	76.4 ± 1.3 ^b^	30.8 ± 0.6 ^a^
Rakaposhi Nagar	Agricultural land	20.5 ± 0.1 ^a^	1.45 ± 0.35 ^a^	13.3 ± 0.3 ^b^	97.7 ± 0.7 ^a^	37.9 ± 0.5 ^a^
	Forest land	12.1 ± 0.3 ^c^	0.60 ± 0.14 ^b^	12.4 ± 0.1 ^c^	31.4 ± 1.2 ^d^	12.9 ± 1.8 ^b^
	Pastureland	11.7 ± 0.2 ^c^	0.40 ± 0.14 ^b^	11.7 ± 0.1 ^c^	51.3 ± 1.13 ^c^	11.6 ± 1.8 ^b^
	Permafrost	16.7 ± 0.3 ^b^	1.45 ± 0.07 ^a^	16.1 ± 0.2 ^a^	80.0 ± 0.7 ^b^	30.2 ± 0.6 ^a^
Jutial Gilgit	Agricultural land	21.4 ± 0.1 ^a^	0.8 ± 0.14 ^b^	14.5 ± 0.6 ^a^	91.8 ± 0.4 ^a^	23.3 ± 1.1 ^a^
	Forest land	12.7 ± 0.4 ^c^	0.4 ± 0.14 ^c^	11.3 ± 0.6 ^b^	31.4 ± 0.2 ^d^	12.9 ± 1.9 ^c^
	Pastureland	12.4 ± 0.3 ^c^	0.25 ± 0.21 ^c^	11.5 ± 0.4 ^b^	34.0 ± 0.7 ^c^	20.6 ± 1.1 ^b^
	Permafrost	16.1 ± 0.3 ^b^	1.7 ± 0.14 ^a^	14.9 ± 0.1 ^a^	84.6 ± 0.4 ^b^	21.1 ± 0.8 ^a^

Significant differences in various land uses are shown by letters in superscript.

**Table 4:** Zn concentration in various land uses of Central Karakoram using different extraction methods.

Regions	Land Uses	Zn Concentration Using Different Extraction Methods				
		KNO_3_ (mg/kg)	H_2_O (mg/kg)	NaOH (mg/kg)	Na_2_EDTA (mg/kg)	HNO_3_ (mg/kg)
Gojal Hunza	Agricultural land	121 ± 0.1 ^a^	1.45 ± 0.07 ^a^	17.7 ± 0.1 ^a^	336 ± 1 ^a^	298 ± 1 ^a^
	Forest land	47.1 ± 3.3 ^d^	0.8 ± 0.14 ^b^	7.85 ± 0.3 ^d^	124 ± 1 ^c^	188 ± 0 ^c^
	Pastureland	74.4 ± 0.2 ^b^	0.75 ± 0.07 ^b^	12.9 ± 0.7 ^c^	107 ± 0 ^d^	86.2 ± 0 ^d^
	Permafrost	64.2 ± 0.5 ^c^	1.6 ± 0.14 ^a^	15.9 ± 0.4 ^b^	239 ± 1 ^b^	199 ± 1 ^b^
Karimabad Hunza	Agricultural land	110 ± 0.6 ^a^	1.65 ± 0.06 ^a^	24 ± 0.7 ^a^	304 ± 1 ^a^	299 ± 1 ^a^
	Forest land	67.1 ± 0.2 ^d^	0.45 ± 0.07 ^c^	19.5 ± 0.1 ^b^	120 ± 1 ^c^	131 ± 1 ^c^
	Pastureland	70.0 ± 1.6 ^c^	0.75 ± 0.07 ^c^	19.9 ± 1.3 ^b^	101 ± 2 ^d^	96.5 ± 0.5 ^d^
	Permafrost	79.6 ± 0.3 ^b^	1.05 ± 0.21 ^b^	20.2 ± 0.5 ^b^	246 ± 1 ^b^	146 ± 1 ^b^
Rakaposhi Nagar	Agricultural land	120 ± 0.9 ^a^	1.7 ± 0.14 ^a^	18.8 ± 1.4 ^a^	325 ± 1 ^a^	144 ± 1 ^c^
	Forest land	65.1 ± 1.1 ^c^	0.45 ± 0.07 ^b^	8.0 ± 0.9 ^c^	102 ± 1 ^d^	178 ± 1 ^a^
	Pastureland	87.9 ± 1.4 ^b^	0.35 ± 0.21 ^b^	13.9 ± 0.8 ^b^	106 ± 0 ^c^	93.1 ± 1 ^d^
	Permafrost	87.7 ± 0.2 ^b^	1.2 ± 0.42 ^a^	14.2 ± 0.5 ^b^	221 ± 1 ^b^	169 ± 1 ^b^
Jutial Gilgit	Agricultural land	119 ± 1.6 ^a^	1.2 ± 0.01 ^a^	18.3 ± 0.6 ^a^	325 ± 1 ^a^	268 ± 1 ^a^
	Forest land	68.7 ± 0.3 ^d^	0.55 ± 0.07 ^c^	9.0 ± 0.6 ^c^	108 ± 1 ^c^	169 ± 1 ^d^
	Pastureland	78.5 ± 0.1 ^c^	0.15 ± 0.05 ^c^	13.1 ± 0.5 ^b^	105 ± 1 ^d^	92.6 ± 3 ^a^
	Permafrost	87.5 ± 0.1 ^b^	1.35 ± 0.07 ^a^	14.3 ± 0.9 ^b^	235 ± 0 ^b^	159 ± 1 ^c^

The significant differences in various land uses are shown by the letters in superscript.

**Table 5:** Pearson correlation coefficients for MEs across all four regions of Central Karakoram.

Physio-Chemical Properties	Regions											
	Gojal Hunza			Karimabad Hunza			Rakaposhi Nagar			Jutial Gilgit		
	Cu	Ni	Zn	Cu	Ni	Zn	Cu	Ni	Zn	Cu	Ni	Zn
pH	0.41	0.13	0.02	0.77	0.28	0.09	0.08	−0.24	−0.18	0.79	0.73	0.72
EC	0.99	0.99 **	0.97 *	0.81	−0.16	−0.30	0.75	0.47	0.65	0.17	0.08	0.06
MC	0.23	0.36	0.54	−0.91	0.11	0.30	0.13	0.41	0.16	−0.06	0.15	0.13
OM	−0.47	−0.44	−0.57	0.34	−0.69	−0.75	−0.96 *	−0.96 *	−0.92	−0.52	−0.66	−0.63

**Note:** The numbers in bold represent significant correlations, with n = 4, and *p* = < 0.05 (*), and *p* < 0.01 (**).

## Data Availability

The data presented in this study are available on request from the corresponding author.
